# Acetic Acid Bacteria Genomes Reveal Functional Traits for Adaptation to Life in Insect Guts

**DOI:** 10.1093/gbe/evu062

**Published:** 2014-03-28

**Authors:** Bessem Chouaia, Stefano Gaiarsa, Elena Crotti, Francesco Comandatore, Mauro Degli Esposti, Irene Ricci, Alberto Alma, Guido Favia, Claudio Bandi, Daniele Daffonchio

**Affiliations:** ^1^Department of Food, Environmental, and Nutritional Sciences (DeFENS), University of Milan, Italy; ^2^Dipartimento di Scienze Veterinarie e Sanità Pubblica (DIVET), University of Milan, Italy; ^3^Italian Institute of Technology (IIT), Genoa, Italy; ^4^Scuola di Bioscienze e Biotecnologie, Università degli Studi di Camerino, Camerino, Italy; ^5^Dipartimento di Scienze Agrarie (DISAFA), Forestali e Alimentari, University of Turin, Grugliasco, Italy

**Keywords:** symbiosis, acetic acid bacteria, cytochrome oxidase

## Abstract

Acetic acid bacteria (AAB) live in sugar rich environments, including food matrices, plant tissues, and the gut of sugar-feeding insects. By comparing the newly sequenced genomes of *Asaia platycodi* and *Saccharibacter* sp., symbionts of *Anopheles stephensi* and *Apis mellifera*, respectively, with those of 14 other AAB, we provide a genomic view of the evolutionary pattern of this bacterial group and clues on traits that explain the success of AAB as insect symbionts. A specific pre-adaptive trait, cytochrome *bo*_3_ ubiquinol oxidase, appears ancestral in AAB and shows a phylogeny that is congruent with that of the genomes. The functional properties of this terminal oxidase might have allowed AAB to adapt to the diverse oxygen levels of arthropod guts.

## Introduction

Besides plant tissues and food matrices, acetic acid bacteria (AAB) live in symbiosis with insects (reviewed in Crotti et al. 2010). Several research teams have investigated the relationship between AAB and their host (Crotti et al. 2010) focusing on the insect gut. In addition to the intestine, AAB could also be localized in other insect body compartments. For instance, the acetic acid bacterium *Asaia* colonizes not only the gut but also the salivary glands and the male and female reproductive systems, which are crucial sites for the bacterial transmission by horizontal and vertical routes (Damiani et al. 2008; Crotti et al. 2009; Gonella et al. 2012). Studies aiming to understand the nature of AAB symbiosis focused on the role of or potential advantages given by AAB to their respective hosts (Ryu et al. 2008; Chouaia et al. 2012; Lee et al. 2013). Key traits for intimately interacting with the insect host include, among others, the capacity to colonize host tissues and the interaction with the innate immunity and the developmental pathways of the host (Ryu et al. 2008; Gross et al. 2009; Douglas et al. 2011; Shin et al. 2011; Lee et al. 2013; Login and Heddi 2013). For instance, *Asaia* exerts a beneficial role during the development of mosquito larvae (Chouaia et al. 2012; Mitraka et al. 2013) affecting the expression of genes related to the cuticle formation (Mitraka et al. 2013). In *Drosophila*, AAB are involved in the modulation of innate immunity, which keeps pathogenic strains under control (Ryu et al. 2008). Moreover, in the same host, *Acetobacter pomorum* modulates the insulin signaling, a pathway involved in the regulation of development, body size, energy metabolism, and intestinal stem cell activity of the host (Shin et al. 2011).

There are actually 14 genomes of AAB deposited in the databases but a genomic analysis of the evolutionary factors driving the association with insects is lacking. By including novel genome sequences of two AAB, *Asaia platycodi* and *Saccharibacter* sp., respectively, isolated from the malaria vector *Anopheles stephensi* and the honeybee *Apis mellifera*, we present a genomic evolutionary analysis of AAB for assessing traits associated with the success of some of their members as insect symbionts. We discuss the potential role of alternative terminal oxidases as symbiotic factors favoring the adaptation of AAB to the insect hosts.

## Results and Discussion

### Several Potential Symbiotic Traits Are Present in AAB

Annotation of the *A. platycodi* and *Saccharibacter* sp. genomes revealed a series of traits compatible with a symbiotic life style in the insect gut. *Asaia platycodi* and *Saccharibacter* present several secretion system (Sec-SRP and Tat for both genomes and type IV in the case of *A. platycodi*) and ABC transporters (in the case of *A. platycodi*) that may have roles in the cross talk between the bacterium and the host. A series of bacterial components for motility and cell surface structures can be implicated in the colonization of the gut epithelium by *A. platycodi* and *Saccharibacter*. These include the genes for the flagellar machinery (e.g., *MotA*, *MotB*, *FlaA*, *FlaB*, *FlgC*, *FlgD*, *FlgE2*, *FlgH*, *FtsI*) as well as genes encoding for fimbriae (*sF-Chap* and *sF-UshP*) and glycan biosynthesis. Although these features may help in the establishment of a symbiotic relationship, they are not essential for it. The presence of these traits was not associated with the ability to establish symbiosis. In fact, genes for the flagellar machinery were also present in *Ac. aceti*, *Gluconacetobacter diazotrophicus*, *Gluconobacter frateurii*, *G. morbifer*, *G. oxydans*, and *G. thailandicus* but not *Ac. pomorum* and *Commensalibacter intestini*. This trend was also observed for the other traits.

Both genomes contain the operon for the production of acetoin and 2,3-butandiol: These molecules have been shown to play a role in insects’ pheromone signaling (Tolasch et al. 2003). 2,3-Butandiol has been implicated in the modulation of the innate immunity response of vertebrate hosts, facilitating tissue colonization by pathogenic bacteria (Bari et al. 2011). We can thus speculate that the production of metabolites potentially interfering with insect physiology and innate immunity might have provided AAB with a pre-adaptive feature toward symbiosis with insect hosts. This trait was observed in other AAB including most of those described as insect symbionts (i.e., *Ac. tropicalis*, *C. intestini*, *Glucona. diazotrophicus*, *Glucona. europaeus*, *Glucona. oboediens*, *G. frateurii*, *G. morbifer*, *G. Oxydans*, and *G. thailandicus*).

### Adaptation to Diverse Oxic Conditions

AAB are aerobic organisms, consistent with their lifestyle in oxygen rich environments (Kersters et al. 2006). On the other hand, the oxygen levels in the guts of many arthropods may vary from aerobic to completely anoxic (Sudakaran et al. 2012). We have thus focused our attention on the presence and distribution of the oxygen-reacting systems of the electron transport chain (terminal oxidases). The genomes of both *A. platycodi* and *Saccharibacter* sp. present all the genes for the operons of cytochrome *bo*_3_ (*CyoA-D*) and *bd* (*CydAB*) ubiquinol oxidase, which have high affinity for oxygen. The operons of both cytochrome *bo*_3_ and *bd* oxidase are present in all AAB genomes, often with two different versions for the *bd* oxidase. This implies that both *A. platycodi* and *Saccharibacter* sp., as well as all the other AAB, have the capacity to respire through an aerobic respiratory chain independent from the terminal cytochrome *aa*_3_ oxidase, an enzyme with low affinity for oxygen, that is absent in AAB. Therefore, AAB have the potential to thrive at low oxygen concentrations like the enterobacteria colonizing animal guts, which also do not possess the cytochrome *aa*_3_ oxidase.

**Fig. 1.—:**
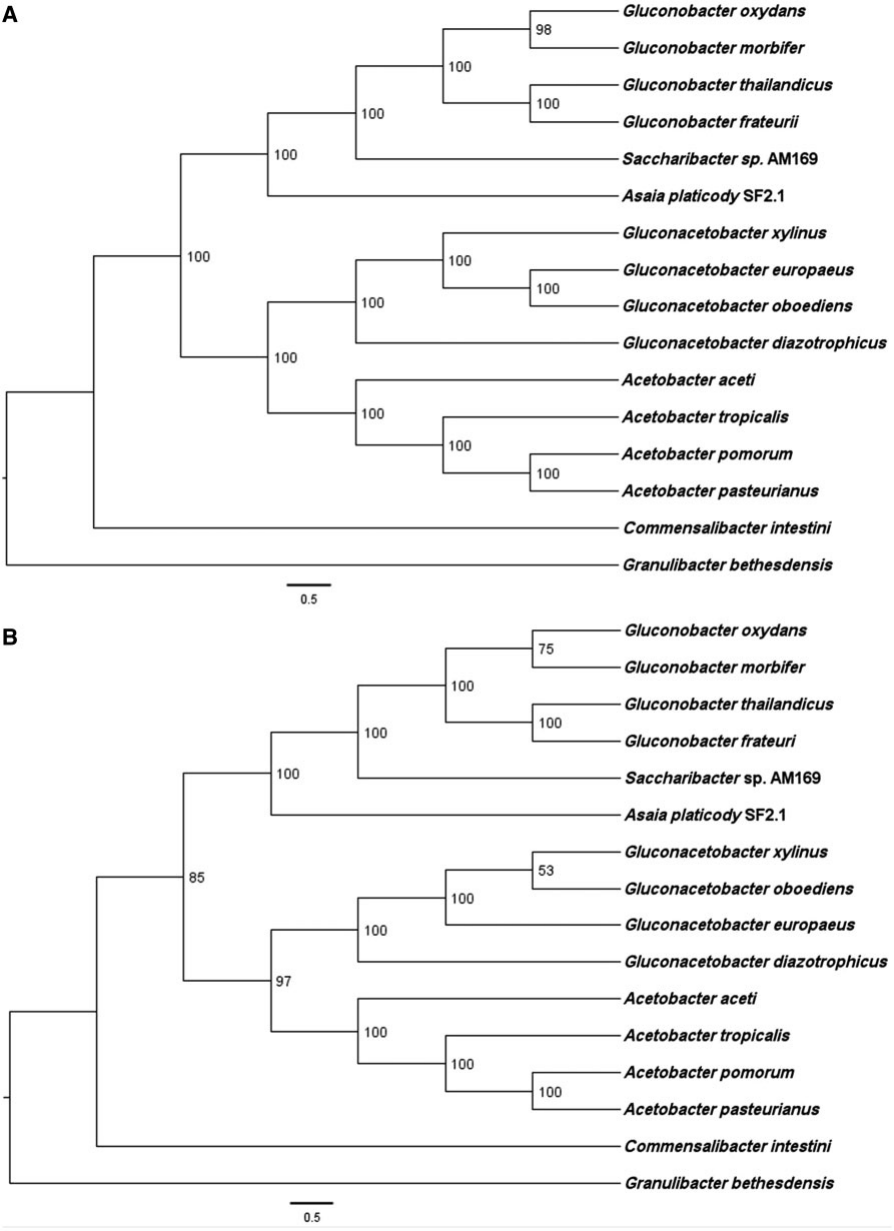
Comparison of species (AAB) (*A*) and operon (ubiquinol oxidase *bo*_3_) (*B*) in phylogenetic trees. The scientific names reported at the terminal nodes are those of the bacterial species. The tree of AAB (top) is based on the results of 70 concatenated protein (supplementary table S1, Supplementary Material online) phylogenetic analyses. Operon tree (bottom) was derived from the phylogeny inferred from the *bo*_3_ operon. The topology shown was obtained by the program RAxML using a partitioned ML model after reconstruction with 1,000 rapid bootstrap.

**Fig. 2.—:**
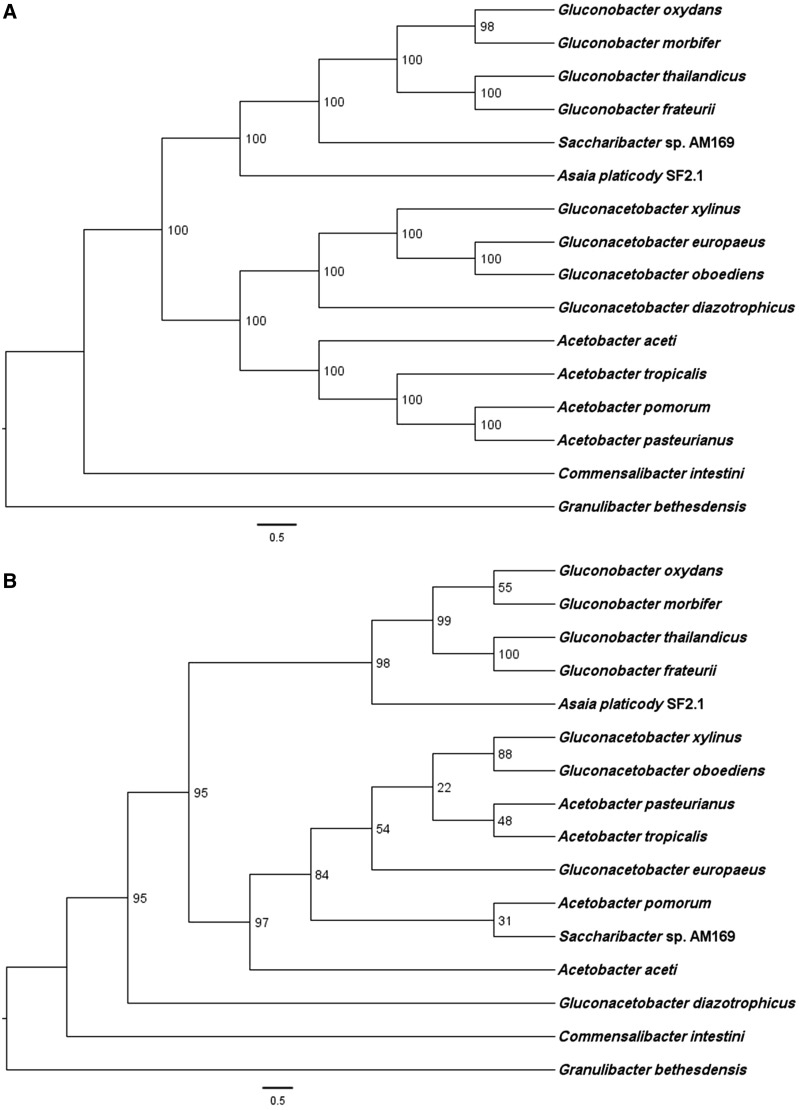
Comparison of species (AAB) (*A*) and operon (cytochrome oxidase *bd*) (*B*) in phylogenetic trees. The scientific names at the terminal nodes are those of the bacteria species. The tree of AAB (top) is based on the results of 70 concatenated protein (supplementary table S1, Supplementary Material online) phylogenetic analyses. Operon tree (bottom) was derived from the phylogeny inferred from the *bd* operon. The tree topology and other details were as given in figure 1.

The phylogenetic tree of the protein subunits of cytochrome *bo*_3_ oxidase of AAB matches that inferred using 70 proteins from the core genome (supplementary table S1, Supplementary Material online, and fig. 1), indicating that *bo*_3_ oxidase evolution followed the differentiation of AAB species from a common ancestor. On the other hand, the phylogenetic tree of the cytochrome *bd* oxidases has a topology that is different from the phylogenomics inferred from core genes (fig. 2). In the *bd* oxidase tree, *Saccharibacter* branches with *Acetobacter* species rather than with *Gluconobacter* (fig. 2) suggesting that lateral gene transfer of *bd* oxidase genes might have occurred along the evolution of AAB.

In sum, although AAB are usually described as strictly aerobic organisms thriving in normoxic environments, our results show that most of these organisms also possess ubiquinol oxidases that should allow their survival under micro-oxic conditions, such as those existing in the insect gut (Sudakaran et al. 2012). Moreover, phylogenetic comparisons show that these terminal oxidases were present in the common ancestor of AAB, thereby constituting an ancestral character. We thus propose that the capacity to thrive at low oxygen concentration conferred by ubiquinol oxidases has provided AAB organisms with a constitutive propensity for thriving in micro-oxic environments including the insect gut, an environment with ample variation in its oxygen levels (Sudakaran et al. 2012). The deep branching of the AAB family contains pathogens such as *Granulibacter bethesdensis* (figs. 1 and 2) further supports that capacity to establish intimate associations with animal hosts is an ancestral trait in these bacteria. On the other hand, the association of AAB with phylogenetically diverse insect species (Crotti et al. 2010) can be considered rather recent, in view of the phylogenetic proximity of symbiont and free-living bacteria (Chouaia et al. 2010).

The analysis of the genomes presented here thus provides new clues indicating ancient pre-adaptation traits to symbiosis in AAB organisms that might have helped, and are still helping, establishing association with insects.

**Fig. 3.—:**
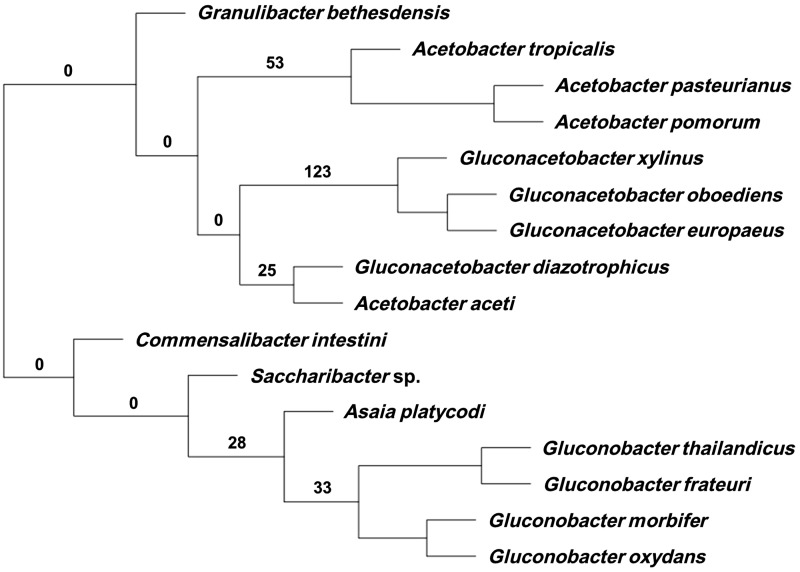
Cluster analysis carried on the total number of ortholog groups after removal of those present in all genomes (i.e., core genome). The analysis shows that groups cluster at the genus level. Numbers on the branches indicate the number of ortholog groups specific to the cluster.

**Fig. 4.—:**
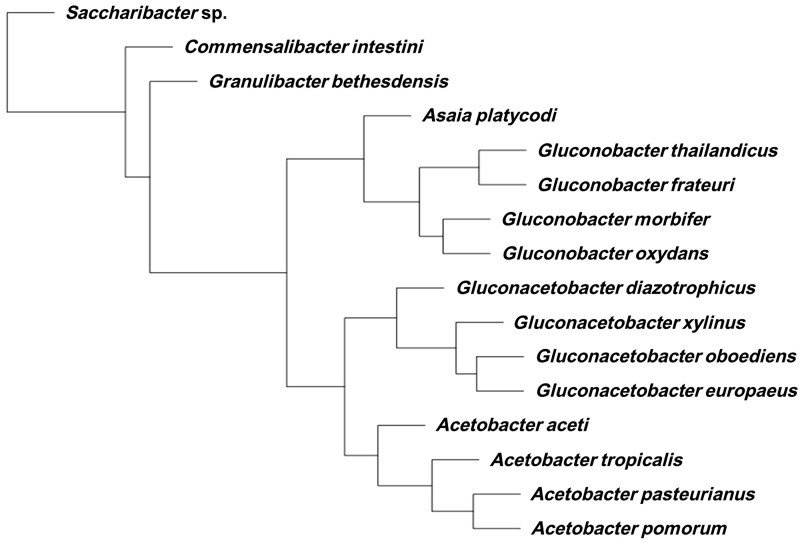
Cluster analysis carried on the subset ortholog groups that were present in at least 50% of the genome. The analysis shows that the clustering of the different groups is congruent with the phylogenomic analysis carried on 70 CDS (fig. 1*A*).

A cluster analysis carried on the ortholog gene groups that were not part of the core genes of the 16 AAB species showed that, in terms of gene acquisitions and losses, there was a coherence at the genus level: All of the members of a given genus clustered together, except for *G. diazotrophicus* and *A. aceti* (fig. 3). However, when only orthologs present in at least 50% of the genomes are considered, the clustering resulting from the analysis of shared genes is congruent to the phylogeny based on the 70 coding sequences (CDS), including the positioning of *G. diazotrophicus* and *A. aceti* (figs. 1*A* and 4). In other words, phenetic analysis based on gene presence/absence produced a tree comparable to that generated by phylogenetics. This result supports the robustness of the phylogenomics here presented for AAB. It is noteworthy that the phylogenomic tree based on 70 CDS was not congruent with 16S rRNA-based phylogeny (supplementary fig. S1, Supplementary Material online)

### Electron Chain Transport and Symbiotic Traits

The analysis of the phylogeny of the cytochrome oxidase operons *bo*_3_ and *bd* showed that both of them were ancestral. The analysis showed also that cytochrome oxidase *bo*_3_ had an evolutionary history similar to that of the AAB genomes. These results suggest that these two operons may have played a role in the symbiotic potential of the AAB.

**Fig. 5.—:**
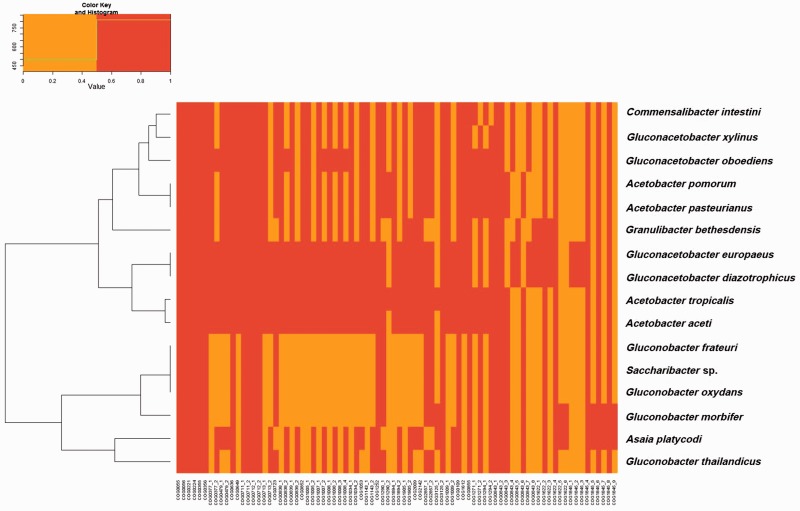
Gene presence–absence analysis of the oxidative phosphorylation chain orthologs in the genomes of AAB. A hierarchical clustering tree (left) was inferred based on the Kulczynski dissimilarity matrix calculated on the presence–absence matrix of genes in the examined genomes. The heatmap to the right of the tree represents the values of the Kulczynski dissimilarity matrix.

In order to investigate the possible implication of other proteins of the electron transport chain in the pre-adaptation of AAB to a symbiotic life, a further cluster analysis was carried on the different orthologs involved in the electron transport chain that were present in the different genomes. This analysis showed that the pattern of gain, loss, and duplication of these genes was coherent and allowed to identify two groups (fig. 5), although there was no correlation between the presence of certain groups of orthologs and the ability to establish symbiosis. The two groups that were identified were the same that emerged from the phylogenomic study. The first group was formed by members of the *Acetobacter* and *Gluconacetobacter* genera, whereas the second group was formed by members of the *Gluconobacter* genus in addition to *Asaia* and *Saccharibacter*.

## Materials and Methods

### Strains

*Asaia platycodi* strain SF2.1 was isolated from *An. stephensi* (Favia et al. 2007). *Saccharibacter* sp. strain AM169 was isolated from an adult gut of *Ap. mellifera* using the pre-enrichment medium ABEM (2.0% d-sorbitol, 0.5% peptone, 0.3% yeast extract pH 3.5; Favia et al. 2007) supplemented with 100 μg ml^−1^ of cycloheximide, followed by a plating on CaCO_3_-containing plates (1.0% d-glucose, 1.0% glycerol, 1.0% ethanol, 1.0% peptone, 0.5% yeast extract, 0.7% CaCO_3,_ and 1.5% agar, pH 6.8). *Saccharibacter* sp. strain AM169 colony was selected based on the capability to clear CaCO_3_. Both strains were characterized as aerobic, Gram-negative, and rod-shaped bacteria belonging to the family Acetobacteraceae.

### Genome Sequencing, Assembly, and Annotation

The whole genome DNAs of *A. platycodi* SF2.1 and *Saccharibacter* sp. AM169 were purified using the DNeasy® Blood and Tissue kit (QIAGEN) and sequenced by Macrogen Korea institute. The genome sequence of *A. platycodi* SF2.1 was determined using a 3-kb paired-end library (∼200 × 10^3^ reads, ∼80 Mb) with the Genome Sequencer FLX system (Roche, Diagnostics, Branford, CT) and a 100-bp library (∼28 × 10^6^ reads, ∼3 Gb) with Genome AnalyzerIIx (Illumina, San Diego, CA). Raw data were assembled into 27 contigs—generated using Mira (version 3.4) (Chevreux et al. 1999); total coverage over the whole genome reached ∼500-fold. The draft genome was 3,420,092 bp in length and contained 3,137 open reading frames (ORFs). The G+C content of the genome was 59.9%. The genome of *Saccharibacter* sp. AM169 was obtained from a tenth if a lane of Illumina Hiseq2000 platform generating 101-pb-long pair-end reads (∼24 × 10^6^ reads, ∼2.4 Gb). The nine contigs were generated using Velvet (version 1.2) (Zerbino and Birney 2008); total coverage over the whole genome reached ∼1000-fold. The draft genome was 1,978,091 bp in length and contained 1,877 ORFs. The G+C content of the genome was 59.3% (table 1).

**Table 1:** General Genome Features of *Asaia platicody* and *Saccharibacter* sp

Organism	*A. platicody* SF2.1	*Saccharibacter* sp. AM169
Genome size (pb)	3,420,092	1,978,091
Number of contigs	27	9
GC%	59.9	59.3
CDS	3,134	1,877
tRNAs	56	58
rRNAs	3	3
Accession number	CBLX010000001:27	CBLY010000001:9
Isolation year	2005	2010

The functional annotation of the predicted genes was performed using the RAST server (Aziz et al. 2008) combined with KEGG (Kanehisa et al. 2004) and COG (Tatusov et al. 2003) databases.

Genomes of *A. platycodi* and *Saccharibacter* sp. were also checked against the genomes of *G. oxydans* and *Glucona. diazotrophicus* using the KEGG database. The search for specific genes was carried out by local BLAST against the different genomes downloaded from the NCBI website using well-characterized and annotated genes. Confirmation or rectification for genes’ annotation was obtained by additional DeltaBLAST analysis (http://blast.ncbi.nlm.nih.gov/Blast.cgi, last accessed April 8, 2014).

The result of this whole-genome project has been deposited at EMBL/GenBank database under the accession numbers CBLX010000001–CBLX010000027 for *A. platycodi* and CBLY010000001–CBLY010000009 for *Saccharibacter* sp. The material described in this article corresponds to the first version of the submitted genomes.

### Phylogenomics and Phylogenetic Reconstruction of Specific Operons

For the phylogenetic reconstruction of the AAB, 14 available (table 2), complete or draft, genomes were downloaded from the NCBI database along with our two genomes. A standardized ORF calling using Prodigal (Hyatt et al. 2010) was performed on all the nucleotidic sequences and nontruncated proteins longer than 50 residues were kept for the following analysis. Orthologs present in a single copy in any given genome were then selected using OrthoMCL (Li et al. 2003) and a custom script designed to keep only those matching with a single or no COG entry. The amino acid sequences of CDS belonging to each ortholog family were aligned using MUSCLE (Edgar 2004); the alignments were subsequently retro-transcribed to their respective nucleic acid sequences, which were checked for the probability of recombination and lateral gene transfer using the phi-test under the Phi-pack (Bruen et al. 2006). At the end of this screening, 70 proteins were kept for phylogenetic analysis (listed in supplementary table S1, Supplementary Material online). The alignment for the remaining CDS was Gblocked, keeping only the proteins that had <3 misaligned residues. For each of the aligned CDS, an evolutionary model was predicted using ProtTest (Darriba et al. 2011); then all the protein sequences were concatenated and their phylogenetic tree was constructed with RAxML (Stamatakis et al. 2005) using a partitioned Maximum Likelihood model that takes into account the evolutionary model predicted for each of the CDS. The phylogenetic trees were tested with 1,000 rapid bootstraps. The phylogenetic trees of the concatenated protein subunits of the cytochrome *bo*_3_ and *bd* oxidases were constructed with the same method. A phylogenetic tree based on 16S rRNA was also inferred (see supplementary material, Supplementary Material online)

**Table 2:** List of Bacterial Genomes Used for the Phylogenetic Studies

Organism	Accession Number	Reference	Origin
*Acetobacter aceti* NBRC 14818	PRJNA70715/PRJDA52649	Sakurai et al. (2011)	Reference strain
*Ac. pasteurianus* IFO 3283-01	PRJNA59279/PRJDA31129	Azuma et al. (2009)	Cocoa bean heap fermentation
*Ac. pomorum* DM001	PRJNA65823/PRJNA60787	Shin et al. (2011)	*Drosophila melanogaster*
*Ac. tropicalis* NBRC 101654	PRJNA68643/PRJDA46891	Matsutani et al. (2011)	Fruits
*Asaia platycodi* SF2.1	CBLX010000001:27	This study	*Anopheles stephensi*
*Commensalibacter intestini* A911	PRJNA75109/PRJNA73359	Roh et al. (2008)	*Drosophila melanogaster*
*Gluconacetobacter diazotrophicus* PAl 5	PRJNA61587/PRJNA377	Bertalan et al. (2009)	Sugarcane plants
*Glucona. europaeus* LMG 18494	PRJNA73763/PRJEA61325	Andrés-Barrao et al. (2011)	Reference strain
*Glucona. oboediens* 174Bp2	PRJNA73765/PRJEA61333	Andrés-Barrao et al. (2011)	Spirit vinegar
*Glucona. xylinus* NBRC 3288	PRJNA46523/PRJDA64985	Ogino et al. (2011)	Vinegar
*Gluconobacter frateurii* NBRC 101659	PRJNA178735/PRJDB2	Hattori et al. (2012)	Reference strain
*G. morbifer* G707	PRJNA76941/PRJNA73361	Roh et al. (2008)	*Drosophila melanogaster*
*G. oxydans* H24	PRJNA179202/PRJNA173388	Ge et al. (2013)	Reference strain
*G. thailandicus* NBRC 3255	PRJDB753/PRJNA191942	Matsutani et al. (2013)	Strawberry
*Granulibacter bethesdensis* CGDNIH1	PRJNA58661/PRJNA17111	Greenberg et al. (2007)	Chronic granulomatous disease patient
*Saccharibacter* sp. AM169	CBLY010000001:9	This study	*Apis mellifera*

### Cluster Analysis on the Orthologs

The data obtained from OrthoMCL (5,488 ortholog groups) were transformed into a matrix reporting the presence of each ortholog group. The orthologs present in all of the genomes (i.e., core genome) were removed from the data set, leaving 4,575 ortholog groups, and a cluster analysis (Murtagh 1985) was carried out using R (cran.r-project.org, last accessed April 8, 2014). A second cluster analysis was carried out on a subset consisting only of the ortholog groups (1,167 ortholog groups) present in at least 50% of the genomes. The pattern of presence/absence was reconstructed also for the subset of genes involved in the oxidative phosphorylation chain. The oxidative phosphorylation chain genes (listed in supplementary table S2, Supplementary Material online) were identified on the basis of KEGG annotation of the *Glucona. diazotrophicus* PAl 5, *G. oxydans* H24, *Ac. pasteurianus* IFO 3283-01, and *Granulibacter bethesdensis* strain genomes; the relative presence/absence informations were retrieved from the OrthoMCL matrix generated above and organized in a new matrix. This new matrix was subjected to hierarchical clustering analysis using the Kulczynski distance index and the heatmap graphic representation was generated with R.

## Supplementary Material

Supplementary methods, tables S1 and S2, and figure S1 are available at *Genome Biology and Evolution* online (http://www.gbe.oxfordjournals.org/).

Supplementary Data
