# Supplementary material for: Acetic Acid Bacteria Genomes Reveal Functional Traits for Adaptation to Life in Insect Guts
Source: Genome Biol Evol. 2014 Mar 28;6(4):912–20. doi: 10.1093/gbe/evu062 (PMC4007555; doi:10.1093/gbe/evu062)
Supplement: Supplementary Data [file supp_6_4_912__index.html]

Acetic acid bacteria genomes reveal functional traits for adaptation to life in insect guts — Acetic Acid Bacteria Genomes Reveal Functional Traits for Adaptation to Life in Insect Guts — Supplementary Data 

# Acetic Acid Bacteria Genomes Reveal Functional Traits for Adaptation to Life in Insect Guts

## Supplementary Data

files

**Files in this Data Supplement:**

- Supplementary Data - doc file
