# Supplementary material for: Acetic Acid Bacteria Genomes Reveal Functional Traits for Adaptation to Life in Insect Guts
Source: Genome Biol Evol. 2014 Mar 28;6(4):912–20. doi: 10.1093/gbe/evu062 (PMC4007555; doi:10.1093/gbe/evu062)
Supplement: Supplementary Data [file supp_evu062_Chouaia_et_al_revised_supplementary_material_06_03_2014.doc]

**Supplementary table 1**: list of genes used for the reconstruction of the phylogeny of the acetic acid bacteria

| Gene | Accession number |
| --- | --- |
| 2-C-methyl-D-erythritol 2,4-cyclodiphosphate synthase | YP_744840 |
| 2-isopropylmalate synthase | WP_008853878 |
| 2-nitropropane dioxygenase | WP_008854734 |
| 3-oxoacyl-ACP synthase | WP_008854808 |
| 4-hydroxy-3-methylbut-2-en-1-yl diphosphate synthase | WP_008855089 |
| 50S ribosomal protein L1 | WP_008853148 |
| 50S ribosomal protein L33 | WP_008853818 |
| ABC transporter ATP-binding protein uup | YP_744566 |
| acetolactate synthase | WP_008853574 |
| alanyl-tRNA synthetase | WP_008853271 |
| argininosuccinate synthase | YP_744084 |
| aspartyl/glutamyl-tRNA amidotransferase subunit A | YP_744773 |
| carbamoyl phosphate synthase large subunit | YP_746109 |
| carbamoyl phosphate synthase small subunit | WP_008853214 |
| cell division protein FtsZ | WP_010503964 |
| chromosomal replication initiation protein | WP_008853748 |
| cysteine desulfarase | WP_008853644 |
| delta-aminolevulinic acid dehydratase | WP_008853705 |
| deoxycytidine triphosphate deaminase | WP_008854881 |
| DNA gyrase subunit B | WP_008853745 |
| DNA helicase II | WP_008853851 |
| DNA polymerase | WP_008854455 |
| DNA polymerase III subunit alpha | WP_008853676 |
| DNA topoisomerase I | WP_008853332 |
| DNA-directed RNA polymerase subunit beta | WP_008853152 |
| elongation factor 4 | WP_008853590 |
| elongation factor Tu | YP_744373 |
| glucosamine--fructose-6-phosphate aminotransferase | YP_745059 |
| glutamyl-tRNA synthetase | YP_744635 |
| GMP synthase | YP_744971 |
| GTP-binding protein TypA | YP_745941 |
| heme ABC transporter ATP-binding protein | WP_008854237 |
| hypothetical protein | WP_008854252 |
| hypothetical protein | WP_008854127 |
| hypothetical protein | WP_008853216 |
| hypothetical protein | WP_008854002 |
| hypothetical protein GbCGDNIH1_1105 | YP_744926 |
| hypothetical protein GbCGDNIH1_2104 | YP_745925 |
| hypothetical protein GbCGDNIH1_2262 | YP_746083 |
| isocitrate dehydrogenase | YP_001602236 |
| lipoprotein releasing system transmembrane protein lolE | YP_745104 |
| lysyl-tRNA synthetase | WP_008854093 |
| methionine aminopeptidase | YP_745474 |
| modulator protein | WP_008854126 |
| molecular chaperone DnaK | WP_008854555 |
| molybdenum cofactor biosynthesis protein A | YP_746227 |
| N-acetylglutamate synthase | WP_008853484 |
| NAD synthetase | YP_744108 |
| nucleoside-diphosphate-sugar epimerases | YP_745799 |
| organic solvent tolerance protein | YP_746018 |
| peptide chain release factor 3 | YP_744350 |
| phosphoribosylaminoimidazole-succinocarboxamide synthase | WP_008853798 |
| phosphoribosylformylglycinamidine synthase | WP_008851252 |
| polynucleotide phosphorylase | YP_746172 |
| protein ecsC | YP_745666 |
| putative glycerol-3-phosphate acyltransferase PlsX | YP_744834 |
| radical SAM superfamily protein | YP_746222 |
| S-adenosyl-L-homocysteine hydrolase | YP_743885 |
| seryl-tRNA synthetase | WP_008854896 |
| signal recognition particle, subunit FFH/SRP54 | YP_745431 |
| superoxide dismutase | WP_008854904 |
| transcription termination factor Rho | WP_008854582 |
| transcription-repair coupling factor | WP_008853368 |
| transcriptional regulator | YP_006984873 |
| translocation protein TolB | YP_744927 |
| trigger factor | YP_745130 |
| tRNA pseudouridine synthase B | WP_008854731 |
| ubiquinone/menaquinone biosynthesis methyltransferase | WP_008853751 |
| UDP-N-acetylmuramoyl-L-alanyl-D-glutamate--2,6-diaminopimelateligase | WP_008853091 |
| valyl-tRNA synthetase | WP_008853757 |

**Supplementary table 2**: list of COGs used in the cluster analysis of the acetic acid bacteria

| COG Id | COG function |
| --- | --- |
| COG0055 | F0F1-type ATP synthase, beta subunit |
| COG0056 | F0F1-type ATP synthase, alpha subunit |
| COG0221 | Inorganic pyrophosphatase |
| COG0224 | F0F1-type ATP synthase, gamma subunit |
| COG0355 | F0F1-type ATP synthase, epsilon subunit (mitochondrial delta subunit) |
| COG0356 | F0F1-type ATP synthase, subunit a |
| COG0377 | NADH:ubiquinone oxidoreductase 20 kD subunit and related Fe-S oxidoreductases |
| COG0479 | Succinate dehydrogenase/fumarate reductase, Fe-S protein subunit |
| COG0636 | F0F1-type ATP synthase, subunit c/Archaeal/vacuolar-type H+-ATPase, subunit K |
| COG0649 | NADH:ubiquinone oxidoreductase 49 kD subunit 7 |
| COG0711 | F0F1-type ATP synthase, subunit b |
| COG0712 | F0F1-type ATP synthase, delta subunit (mitochondrial oligomycin sensitivity protein) |
| COG0713 | NADH:ubiquinone oxidoreductase subunit 11 or 4L (chain K) |
| COG0723 | Rieske Fe-S protein |
| COG0838 | NADH:ubiquinone oxidoreductase subunit 3 (chain A) |
| COG0839 | NADH:ubiquinone oxidoreductase subunit 6 (chain J) |
| COG0843 | Heme/copper-type cytochrome/quinol oxidases, subunit 1 |
| COG0852 | NADH:ubiquinone oxidoreductase 27 kD subunit |
| COG1005 | NADH:ubiquinone oxidoreductase subunit 1 (chain H) |
| COG1007 | NADH:ubiquinone oxidoreductase subunit 2 (chain N) |
| COG1008 | NADH:ubiquinone oxidoreductase subunit 4 (chain M) |
| COG1034 | NADH dehydrogenase/NADH:ubiquinone oxidoreductase 75 kD subunit (chain G) |
| COG1053 | Succinate dehydrogenase/fumarate reductase, flavoprotein subunit |
| COG1143 | Formate hydrogenlyase subunit 6/NADH:ubiquinone oxidoreductase 23 kD subunit (chain I) |
| COG1252 | NADH dehydrogenase, FAD-containing subunit |
| COG1271 | Cytochrome bd-type quinol oxidase, subunit 1 |
| COG1290 | Cytochrome b subunit of the bc complex |
| COG1294 | Cytochrome bd-type quinol oxidase, subunit 2 |
| COG1622 | Heme/copper-type cytochrome/quinol oxidases, subunit 2 |
| COG1845 | Heme/copper-type cytochrome/quinol oxidase, subunit 3 |
| COG1894 | NADH:ubiquinone oxidoreductase, NADH-binding (51 kD) subunit |
| COG1905 | NADH:ubiquinone oxidoreductase 24 kD subunit |
| COG2009 | Succinate dehydrogenase/fumarate reductase, cytochrome b subunit |
| COG2142 | Succinate dehydrogenase, hydrophobic anchor subunit |
| COG2857 | Cytochrome c1 |
| COG3125 | Heme/copper-type cytochrome/quinol oxidase, subunit 4 |
| COG1009 | NADH:ubiquinone oxidoreductase subunit 5 (chain L)/Multisubunit Na+/H+ antiporter, MnhA subunit |
| COG1612 | Uncharacterized protein required for cytochrome oxidase assembly |
| COG0474 | Cation transport ATPase |
| COG0855 | Polyphosphate kinase |

**Supplementary methods**

**16S rRNA based phylogeny**

Fifty-eight 16S rRNA sequences, longer than 1400bp, belonging to members of the Acetobacteraceae, were downloadede from the NCBI database when available or taken from the sequenced genomes. The sequences were aligned using Clustal keeping default parameter. Two sequences belonging to Wolbachia sp. were chosen as outgroup. Jmodeltest (Posada, 2009) was used to infer the best nucleotide substitution models using the Akaike Information Criterion (AIC).The resulting alignment was used to infer the phylogenetic relationships of the different organisms using a Maximum likelihood (ML) approach. The ML analysis was performed using the Generalised time-reversible (GTR) model, and deletions were not taken into consideration. The statistical robustness of the clusters was evaluated by bootstrap analysis after 1000 replications (Tamura et al., 2011)

Supplemtary figure S1


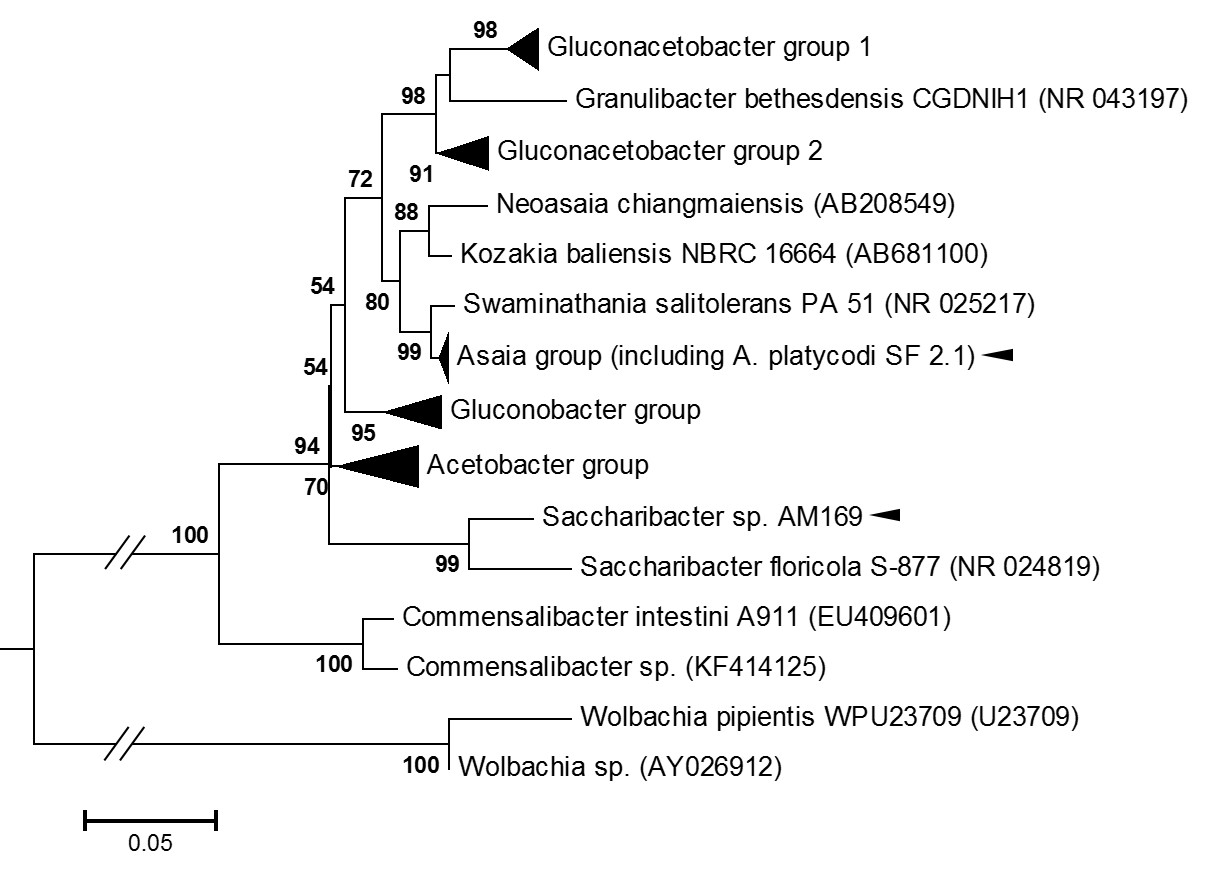


**Figure S1**: Phylogenetic positions of the AAB strains based 16S rRNA gene sequences (Maximum likelihood; GTR model; all positions containing gaps and missing data were eliminated from the data set). Numbers at each node represent the bootstrap percentages of replications calculated from 1,000 replicated trees. The scale bar represents sequence divergence. Grouping was done at the genus level when the cluster was represented by more than five sequences. The position of the sequenced genomes of both *Asaia platycodi* SF2.1 and *Saccharibacter* sp. AM169 is indicated by an arrowhead.

Supplementary references

1. Posada D. 2009 Selection of models of DNA evolution with jModelTest. Methods Mol Biol. 537:93-112.

2. Tamura K, Peterson D, Peterson N, Stecher G, Nei M and Kumar S. 2011. mega5: molecular evolutionary genetics analysis using maximum likelihood, evolutionary distance, and

maximum parsimony methods. Molecular Biology and Evolution. 28:2731–2739.
